# Is It Possible to Differentiate Chronic Kidney Disease and Preeclampsia by means of New and Old Biomarkers? A Prospective Study

**DOI:** 10.1155/2015/127083

**Published:** 2015-10-08

**Authors:** Alessandro Rolfo, Rossella Attini, Elisabetta Tavassoli, Federica Vigotti Neve, Marco Nigra, Matteo Cicilano, Anna Maria Nuzzo, Domenica Giuffrida, Marilisa Biolcati, Michele Nichelatti, Pietro Gaglioti, Tullia Todros, Giorgina Barbara Piccoli

**Affiliations:** ^1^Department of Surgical Sciences, O.I.R.M-Sant'Anna Hospital, University of Turin, Via Ventimiglia 3, 10126 Turin, Italy; ^2^SS Nephrology, Department of Clinical and Biological Sciences, San Luigi Gonzaga Hospital, University of Turin, 10043 Orbassano, Italy; ^3^Chemical-Clinical and Microbiological Analysis Laboratory, Giovanni Bosco Hospital, 10154 Turin, Italy; ^4^Service of Biostatistics, Department of Hematology, Niguarda Ca' Granda Hospital, 20162 Milan, Italy

## Abstract

*Objective*. Chronic kidney disease (CKD) and preeclampsia (PE) may both present with hypertension and proteinuria in pregnancy. Our objective is to test the possibility of distinguishing CKD from PE by means of uteroplacental flows and maternal circulating sFlt-1/PlGF ratio.* Design*. Prospective analysis.* Population*. Seventy-six patients (35 CKD, 24 PE, and 17 other hypertensive disorders), with at least one sFlt-1/PlGF and Doppler evaluation after the 20th gestational week.* Methods*. Maternal sFlt-1-PlGF were determined by immunoassays. Abnormal uterine artery Doppler was defined as resistance index ≥ 0.58. Umbilical Doppler was defined with gestational-age-adjusted Pulsatility Index. Clinical diagnosis was considered as reference. Performance of Doppler study was assessed by sensitivity analysis; sFlt-1/PlGF cut-off values were determined by ROC curves.* Results*. The lowest sFlt-1/PlGF ratio (8.29) was detected in CKD, the highest in PE (317.32) (*P* < 0.001). Uteroplacental flows were mostly preserved in CKD patients in contrast to PE (*P* < 0.001). ROC analysis suggested two cut-points: sFlt-1/PlGF ≥ 32.81 (sensitivity 82.93%; specificity 91.43%) and sFlt-1/PlGF ≥ 78.75 (sensitivity 62.89%, specificity 97.14%). Specificity reached 100% at sFlt-1/PlGF ≥ 142.21 (sensitivity: 48.8%). Early-preterm delivery was associated with higher sFlt-1/PlGF ratio and abnormal uteroplacental flows relative to late-preterm and term deliveries.* Conclusions*. sFlt-1/PlGF ratio and uteroplacental flows significantly correlated with PE or CKD and preterm delivery.

## 1. Introduction 

Preeclampsia (PE) and chronic kidney disease (CKD) in pregnancy share several features. Under these broad and recently changed definitions there are both mildly affected subjects compatible with term delivery of appropriate for gestational age babies and stormy, severe diseases that result in small for gestational age (SGA) babies with relevant morbidity and mortality [1–4].

CKD has been redefined in the new millennium as any pathological abnormality or marker of damage, including abnormalities in blood or urine tests or in imaging studies, irrespective of kidney function (which is used for classification), lasting for at least 3 months [5]. According to these broad definitions, PE and CKD have a similar prevalence, being encountered in 3–5% of all pregnancies [1–4, 6].

The commonly held definition of PE states that it is a reversible condition characterized by hypertension and proteinuria, occurring after the 20th week of pregnancy in previously normotensive, nonproteinuric women [7–10]. More recently, the ACOG guidelines state that PE may be diagnosed in the absence of proteinuria, when new-onset hypertension is accompanied by one of the following: serum creatinine increase, low platelet count or high liver enzymes, pulmonary edema, or central symptoms [11]. The kidney is central to these definitions; the broad etiquette of “superimposed PE” has been used to indicate both placental dysfunction and worsening of the kidney disease, adding to the current ambiguity of this term [12]. Regardless of the definition, proteinuria reflects damage to the glomerular filtration barrier, mainly reflected by podocyturia, which may be a first sign of permanent renal damage [13, 14].

Since both PE and CKD are characterised by proteinuria, hypertension, and progressive renal impairment, the differential diagnosis may be difficult or impossible during pregnancy, and occasionally also after delivery [15, 16].

Two previous retrospective analyses by our group suggested a role for uteroplacental flows and for two serum biomarkers, soluble Fms-like tyrosine kinase 1 (sFlt-1) and Placental Growth Factor (PlGF), in distinguishing between PE and chronic kidney disease (CKD) in patients with hypertension and proteinuria during pregnancy [16, 17].

The interest for the uteroplacental flow analysis is linked to its role not only in the diagnosis, but also in the clinical management of PE patients; the low precision is counterbalanced by the wide availability and simplicity of use [18–20]. The sFlt-1/PlGF ratio is considered one of the most promising biomarkers for preeclampsia. Its use in clinical practice is still controversial [21–26]. In fact, some authors objected that sFlt-1/PlGF ratio best correlated with overt PE, whose diagnosis is usually self-evident, and that, in the absence of overt PE, the predictive value for PE development is not precise enough to be added to the routine clinical work-up [24–29].

The objective of the present prospective study is testing a different indication for both Doppler study and serum biomarkers: the possibility to distinguish between CKD and PE by adding the uteroplacental flow analysis and sFlt-1/PlGF ratio to the clinical definitions. This is the first prospective study to combine the sFlt-1/PlGF ratio with uteroplacental flow for the differential diagnosis of CKD versus PE, taking into account also other related hypertensive pregnancy disorders.

## 2. Methods

### 2.1. Study Population

The study was conducted at the Gynaecology and Obstetrics Unit of the O.I.R.M-Sant'Anna Hospital, University of Turin (Turin, Italy). The study was performed in adherence to the Declaration of Helsinki. Patients were recruited and blood samples were collected after obtaining informed consent, in accordance with the ethics guidelines of the O.I.R.M-Sant'Anna Hospital Ethics Committee (approval of the Ethics Committee of O.I.R.M.-Sant'Anna Hospital number 335; protocol 11551/c28.2; 4/3/2011).

The study consecutively enrolled seventy-six patients with known CKD (*n* = 35) or with a clinical suspicion of PE (PE *n* = 24; other hypertensive disorders *n* = 17) who had the following characteristics in the period January–December 2013.

#### 2.1.1. CKD

Patients were followed up in the Day Hospital-Day Service of our Obstetrics Unit with a diagnosis of CKD antedating pregnancy, hypertension, and/or proteinuria >0.3 g/day. CKD was defined as “any anomaly of blood and urine composition, or imaging or pathological data, lasting for at least three months or a Glomerular Filtration Rate (GFR) below 60 mL/min for the same time period” [5]. GFR was calculated by the Cockroft-Gault and MDRD formulae or by CKD-EPI formula on data collected within 3 months prior to conception. CKD-EPI was finally chosen for calculation because of its wider diffusion in nephrology. When this parameter was not available, serum creatinine measured at first control during pregnancy was used, as previously described [30, 31].

#### 2.1.2. Preeclampsia

PE was defined as hypertension and proteinuria >300 mg/day occurring after the 20th gestational week in a previously normotensive, nonproteinuric woman. Both alterations should be reversible within 3 months from delivery [1–3].

Patients with a clinical suspicion of PE were hospitalized in the Gynaecology and Obstetrics Unit; based on the diagnostic criteria, diagnosis was completed only after delivery, according to the criteria mentioned above. Patients were stratified into confirmed PE or “Other” cases (isolated pregnancy-induced hypertension, fetal growth restriction, chronic hypertension, and isolated pregnancy-induced proteinuria).

Exclusion criteria were twin pregnancies, congenital malformations, chromosomal anomalies, or maternal diseases other than CKD and hypertension.

Only patients who delivered a singleton and who had at least one Doppler analysis and one assessment of sFlt-1/PlGF ratio after the 20th gestational week were considered.

### 2.2. sFlt-1 and PlGF Assays

Two mL of venous blood were collected from each patient and control using serological vials without anticoagulant and gel. Serum was obtained by centrifugation at 3000 rpm at 4°C for 20 minutes within 3 h from collection and stored at −80°C until testing. All samples were analyzed at the same time. sFlt-1 and PlGF serum levels were determined in parallel by specific, commercially available electrochemiluminescence immunoassays (Elecsys, Roche, Penzberg, Germany) using a Cobas-e-411 immunoanalyzer following the manufacturer's instructions. The sFlt-1 and PlGF serum levels of our control pregnancies were comparable to those previously reported by Verlohren and colleagues [24, 32].

When two or more samples were available, the first one was chosen.

### 2.3. Doppler Flow Studies

The evaluation of uterine arteries took into account the resistance index (RI) or Pourcelot ratio, defined as peak systolic flow minus peak end diastolic flow divided by peak systolic flow.

According to the literature, an abnormal uterine artery Doppler FVW was defined as the mean (of the two uterine arteries) resistance index (RI) of ≥0.58 [33].

Umbilical artery Doppler waveforms were analyzed using the Pulsatility Index (PI), defined as peak systolic flow minus end diastolic flow divided by mean flow. Normal PI values were defined according to the gestational age-adjusted data proposed by Guiot et al. [34].

### 2.4. Statistical Analysis

Besides uteroplacental flow measurements and sFlt-1/PlGF ratio, the following clinical and laboratory data were gathered: age, parity, race, week of tests, 24-hour proteinuria, serum creatinine, GFR, total serum albumin, blood pressure and antihypertensive therapy, BMI, gestational age at delivery, type of delivery, clinical complications in the mother, fetal weight percentile according to the international birth weight references (InES charts [35]), Apgar index, sex, admission to neonatal intensive care unit, and outcome.

A descriptive analysis was performed as appropriate (mean and standard deviation for parametric data and median and range for nonparametric data, after checking distribution by visual inspection and Shapiro-Wilk test, if needed).

Comparisons among groups were done by ANOVA, which, if significant, was followed by pairwise analysis using the Bonferroni method. Kruskal-Wallis and Mann-Whitney tests were used for nonparametric data. Pearson's *χ* ^2^ was used for categorical variables, after cross-tabulation.

The clinical diagnosis, according to the criteria mentioned above, was considered as the gold standard for reference.

The diagnostic yield of the sFlt-1/PlGF ratio was analysed by ROC curve tracing, followed by Youden's J statistics to determine optimal cut-off values for the sFlt-1/PlGF ratio. The diagnostic yield of the Doppler study was assessed by sensitivity analysis. ROC curves were built considering CKD versus conditions “other than CKD” (PE and other hypertensive disorders or PE alone). The AUC built for the two tests was compared by means of *z* test according to Henley and McNeil.

Significance was assumed when *P* < 0.05, unless dealing with multiple pairwise comparisons: in that case, the significance limit was calculated according to Bonferroni.

## 3. Results 

### 3.1. Clinical Data

Table 1 reports the main characteristics of the study population, recruited from January to December 2013, as assessed at the beginning of pregnancy (age, BMI, and parity) or at the time of testing (kidney function, hypertension, and proteinuria).

From a clinical-biochemical point of view, there was, as expected, a consistent overlap among the three groups. This confirmed the impossibility to distinguish among these conditions solely on the basis of kidney function assessment or proteinuria.

Table 2 reports the main pregnancy-related outcomes in the three groups. Patients with overt PE had a higher incidence of preterm (<37 completed gestational weeks) and early preterm (<34 completed gestational weeks) delivery, lower birth weight, and severe growth restriction (higher prevalence of babies below the 5th centile for gestational age). In contrast, CKD patients, likely also as a reflection of the selection that included many subjects with normal kidney function and milder clinical anomalies, displayed the most favourable outcomes, while the group defined as “Other” showed an intermediate prognosis.

### 3.2. Molecular and Biophysical Biomarkers

Table 3 summarizes biomarker distribution as considered in the three subsets of patients. The test was performed a median of two weeks earlier in CKD patients, as a reflection of the earlier referral of women with known kidney disease. The difference was not statistically significant (Table 1).

In all cases, the clinical definition was taken as a reference.

The lowest sFlt-1/PlGF ratio was detected in CKD patients, while the highest was observed in PE patients on account of the lower sFlt-1 and higher PlGF in the first subset of cases.

The sFLt-1 levels (median: 1893 pg/mL) of CKD patients were in line with the normal ranges for gestational age provided by the analytical kit (median: 1934 pg/mL). In contrast, PlGF levels were somehow lower in CKD patients (median: 270 pg/mL) as compared to normal controls provided by the company (median: 439 pg/mL).

**Figure 1:**
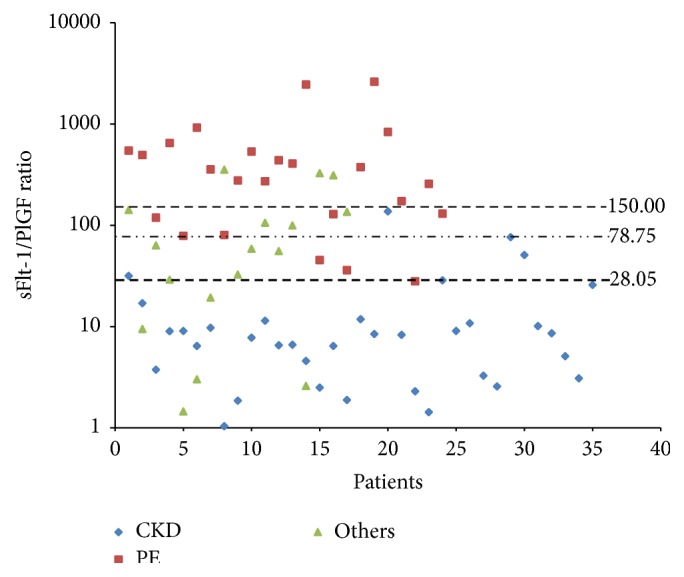
sFlt-1/PlGF ratio distribution pattern in CKD (blue), PE (red), and “other hypertensive disorders” (green) patients.

Figure 1 shows the sFlt-1/PlGF ratio pattern in the three subsets of patients, defined upon the reference clinical criteria. PE patients show the highest ratio, with a minimal overlap with CKD patients, while the “Other” diseases consistently overlap with both PE and CKD. In line with normal placental vascularization, the uteroplacental flow was significantly better preserved in most CKD patients (85.7% normal flows, with no case showing an impairment of both uterine and umbilical Doppler indexes), in stark contrast to PE cases in which only one-third of the subjects (37.5%) had normal Doppler flow indexes at diagnosis. Again, the “other diseases” pattern was intermediate (Table 3).

Taking the clinical diagnosis as reference, sensitivity, specificity, and positive and negative predictive values of Doppler study were calculated considering the presence of at least one impaired uteroplacental flow as indicative for the diagnosis of PE and “Other” diseases versus CKD: sensitivity 56.1% (95% CI: 39.7%–71.5%) and specificity 85.7% (95% CI: 69.7%–95.2%), with a positive predictive value of 82.1% (95% CI: 63.1%–93.9%) and a negative predictive value of 62.5% (95% CI: 47.4%–76.0%).

The diagnostic yield increases considering only the two clinical subsets of PE and CKD: sensitivity 62.5% (95% CI: 40.6%–81.2%) and specificity 85.7% (95% CI: 69.7%–95.2%), with a positive predictive value of 75% (95% CI: 50.9%–91.3%) and a negative predictive value of 76.9% (95% CI: 60.7%–88.9%).

### 3.3. ROC Analysis

The diagnostic potential of the sFlt-1/PlGF ratio versus the clinical diagnosis of CKD or PE was further assessed by ROC curves.

**Figure 2:**
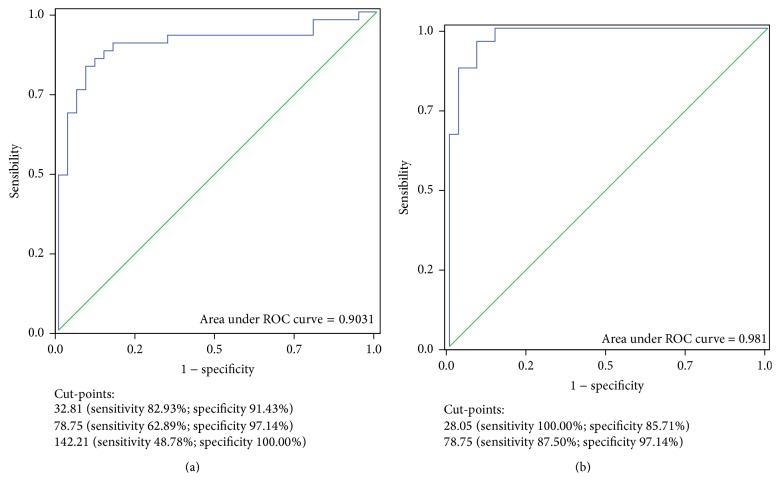
(a) ROC curve obtained considering all cases (*n* = 76); (b) ROC curve obtained by selecting only CKD and PE patients (*n* = 59) and excluding the “Other” diseases.

Figure 2 combines two ROC curves that were prepared taking all cases into consideration (a) and by selecting only the two best defined CKD and PE subsets (b). The ratio was considered for the differential diagnosis between CKD and PE or between CKD and PE plus “Other” diseases in both populations: in other terms, the curves analyse the probability of having a “disease other than CKD,” as clinically defined.

The area of the first curve (all cases: 76 observations) was 0.9031 (Figure 2).

The “best cut-point” identified by Youden's J statistics was at, or above, 32.81 (sensitivity 82.9%; specificity 91.4%; correctly classified: 86.8%). A specificity of 97.1% was obtained at a ratio of 78.75 (sensitivity 62.9%; specificity 97.1%; correctly classified: 86.8%), while specificity reached 100% at or above a ratio of 142.21 (sensitivity: 48.8%; specificity: 100%; correctly classified: 72.4%).

The area under the second ROC curve increased to 0.9810 by selecting clinical criteria only CKD and PE patients (59 cases) and excluding the “Other” diseases. In this analysis, two “best cut-points” may be identified by Youden's J statistics. The first one was at 28.05 (sensitivity 100%; specificity 85.7%; correctly classified: 91.5%), the second one, shared by the first curve, at 78.75 (sensitivity 87.5%; specificity 97.1%; correctly classified: 93.2%).

There was a very close, although incomplete, correlation among flow patterns, sFlt-1, PlGF levels, and their ratio, as shown in Table 4. The median ratio sFlt-1/PlGF was 9.94 in cases with normal umbilical and uterine flows, 136.26 when one arterial flow was altered, and 407.68 when both flows were impaired.

However, the range was wide in all subsets (normal flows: min: 1.04; max: 439.37; both altered flows: min: 9.51; max: 2619.11), with consistent overlap (Table 4).

The accuracy of the ratio sFlt-1/PlGF in identifying diseases other than PE and CKD is higher than that of the flow patterns, the first allowing to correctly classify up to 93% of the cases (limiting the comparison to CKD versus PE, cut-point at ratio 78.75), versus 76.2% in the case of altered flow(s). The difference between the two AUCs (at least one altered flow in the differential diagnosis between CKD and PE: 0.7411 ± 0.0587, versus cut-point at ratio 78.75: 0.9232 ± 0.0373) is significant (*z* test according to Hanley and McNeil: *P* = 0.0088).

### 3.4. Relationship between Biomarkers and Outcome

Regardless of the clinical diagnosis (PE, CKD, or others), a higher ratio and the presence of impaired uteroplacental flow were significantly associated with preterm delivery and SGA babies: the median sFlt-1/PlGF ratio was 9.07 (min 1.04; max 137.40) in the 31 patients who delivered at or after 37 weeks; it was 28.59 (1.86–356.58) in the cases who delivered at 34–37 weeks and 376.21 for patients who delivered before 34 weeks, once more with a very wide dispersion (min–max: 3.09–2619.11) (*P* < 0.001). In line with these data, the median ratio was significantly lower in pregnancies leading to appropriate for gestational age babies (ratio AGA: 19.39 versus SGA: 137.40, *P* = 0.017).

## 4. Discussion

The main result of our study is to suggest that the analysis of sFlt-1 and PlGF may add useful information to the routine clinical assessment of patients with proteinuria and hypertension occurring or first discovered in pregnancy. In this series, an sFlt-1/PlGF ratio above 150 was not compatible with CKD alone, while a ratio below 30 was almost pathognomonic of CKD (Tables 3 and 4).

The study of uteroplacental flows, readily available for a first “bedside” indication, has a good, but incomplete, correlation, as witnessed by relatively low sensitivity and specificity (Table 3).

The clinical definition of the three main subsets of patients emerges as a crucial point: our data are in line with the conclusion of a recent reappraisal of serum biomarkers in “high-risk” pregnancies: “multiple pathogenic pathways lead to the disease recognized clinically as preeclampsia” [27]. The higher precision of ROC curves obtained by including only the cases with a clear clinical diagnosis of PE and CKD, omitting “Other” diseases, emphasizes the importance of a careful clinical characterization. This observation clarifies the difference between the present prospective and the previous retrospective study, in which we excluded the uncertain cases or “Other” diseases considered in the differential diagnosis of PE. While the present study confirms CKD in the previously reported range (ratio below 150), we found higher data variability and a partial overlap between CKD, PE, and “Other” diseases, leading to the suggestion of lowering the discrimination cut-point (Figures 1 and 2).

For the first time to our knowledge, we carried out a prospective attempt to perform differential diagnosis between PE and CKD in pregnancy. The proposed use is to support diagnosis in a setting in which a disease is already present, hence beyond the clinical and ethical limits of “uncertain prediction” in forecasting PE. On the contrary, indication from biomarker analysis may have an immediate clinical implication, since the management of CKD and PE patients may not be the same [30, 31]. However, there are some weaknesses in the present study that may lead to further future analyses. First of all, the study population size is small. Moreover, we did not repeat samples for most of the patients, and in particular for those with PE. In fact, PE patients were usually referred to our tertiary care center in presence of a full-blown clinical picture and usually required rapid delivery. A further limit is represented by the different gestational age at testing, which was lower in CKD mothers, albeit not significantly. Normalization for gestational age should be considered in larger series.

Furthermore, no CKD patient included in our study had the clinical hallmarks of PE superimposed on CKD. Hence, the present study is not able to give insights on this situation, of great potential interest, that should be analysed by means of large, multicentre studies, on prospectively followed CKD patients.

The working hypothesis of our study was that the pathogenesis of proteinuria and hypertension, occurring or worsening in pregnancy in CKD patients, differed from the same signs and symptoms occurring in PE. The “CKD placenta” was expected to develop normally, thus leading to a normal angiogenic/antiangiogenic balance and to normal uteroplacental flows. The use we propose for the sFlt-1/PlGF ratio herein differs from its original indication. This molecular tool was developed in order to predict PE onset in low-risk and high-risk women, a controversial issue due to the imperfect prognostic power and to the risk of clinical interference with the delicate management of high-risk pregnancies [27–29, 36, 37]. Some authors maintain that PE is a self-evident disease and that there is no need for further diagnostic tools. This is probably true in most cases in which prepregnancy data are available. However, the clinical definition cannot cover at least three specific situations involved in the differential diagnosis with CKD. The first one is the lack of data before pregnancy or within the first twenty weeks of gestation: since kidney diseases are frequently asymptomatic, the lack of data hinders differential diagnosis [16, 30, 31]. In our experience, CKD is diagnosed, first encountered, or recognised in its potential clinical importance in over one-third of the cases during pregnancy [30].

The second situation is the onset of a kidney disease during pregnancy. There is no way to differentiate a newly occurring glomerulonephritis from PE by using routine clinical or laboratory tools. This is also true for the third point: the flare during pregnancy of a known glomerulonephritis or immunologic disease that was previously in remission (e.g., systemic lupus erythematosus).

## 5. Conclusions

The differential diagnosis between CKD during pregnancy and PE may be supported both by the analysis of uteroplacental flows and by the analysis of sFlt-1, PlGF, and their ratio. While the flow analysis may be of immediate help, due to the wide bedside availability, there is a major role for sFlt-1 and PlGF in supporting the differential diagnosis.

The data described in this paper may lead to practical clinical suggestions: in pregnancy, the identification of CKD patients may support a more expectant management, while the finding of impaired flows and high ratio may suggest stricter follow-up and support earlier delivery.

After pregnancy, the patients identified with CKD may receive timely care; furthermore, we suggest that particular attention should be posed to the PE cases with low sFlt-1/PlGF ratio and/or normal uteroplacental flows, in order to highlight associated or predisposing diseases that may be more common in this subset of “atypical” cases.

Furthermore, kidney function assessment is not included in the routine work-up of pregnant women according to the main current pregnancy management guidelines, including the Italian ones and the well-known NICE ones [38, 39]. We hope that the growing interest on CKD and pregnancy may help in changing this attitude, leading to the addition of a basic evaluation of the kidney function in all pregnant women.

Further studies are required to refine our findings on a larger scale and to get new physiological insights into the development of proteinuria and hypertension in pregnancy.

The issue of PE superimposed on CKD is of great clinical interest. To analyse this rare situation, we need to set large multicentre studies, prospectively following the patients with repeated sFlt-1 and PlGF measurements since the early stages of pregnancy. The promising results obtained in our series may increase interest and make such analyses possible in the future.

## Figures and Tables

**Table 1:** Main baseline data in the study group.

Data at referral or at 1st test (median; min–max or %)	CKD (*n*: 35)	PE (*n*: 24)	Other (*n*: 17)	Statistical significance
Age (yrs)	32 (19–41)	36.5 (19–54)	36 (28–46)	*P*0 = 0.023
Nulliparous (%)	71.4%	75.0%	47.1%	*P*0 = 0.131
BMI	22.6 (14.8–42.9)	24.9 (14.5–36.0)	27.3 (17.6–43.9)	*P*0 = 0.154
Caucasians (%)	91.4%	95.8%	70.6%	*P*0 = 0.034
Week of test	30 (21–39)	32 (26–36)	34 (25–39)	*P*0 = 0.065
Hypertension (%)	34.3%	100%	100%	*P*0 < 0.001
Proteinuria g/day	0.45 (0.04–3.4)	0.84 (0.3–16.2)	0.23 (0.06–3.7)	*P*0 < 0.001
Proteinuria <0.3 (%)	40%	—	64.7%	*P*0 = 0.004
≥0.3–<0.5 g/day (%)	14.3%	29.2%	11.8%	
0.5–1 g/day (%)	11.4%	25.0%	11.8%	
1–3 g/day (%)	28.6%	29.2%	5.9%	
≥3 g/day (%)	5.7%	16.7%	5.9%	
s-Creatinine (mg/dL)	0.60 (0.38–2.59)	0.67 (0.44–0.99)	0.57 (0.34–0.83)	*P*0 = 0.106
GFR (mL/min)	119 (23–147)	115 (48–144)	124 (91–138)	*P*0 = 0.217

CKD: chronic kidney disease; PE: preeclampsia; BMI: body mass index; GFR: glomerular filtration rate (by CKD-EPI formula); *P*0: significance across groups; *P*1: CKD versus PE; *P*2: CKD versus Other; *P*3: PE versus Other. Significance between groups: maternal age: *P*1 = 0.050; *P*2 = 0.011; *P*3 = 0.62; hypertension: *P*1 < 0.001; *P*2 < 0.001; proteinuria: *P*1 = 0.011; *P*2 = 0.165; *P*3 < 0.001; proteinuria (%): *P*1 = 0.006; *P*2 = 0.368; *P*3 < 0.001.

*Note*. Causes of CKD: glomerular 11; interstitial 13; single kidney 3; diabetic nephropathy 3; Other 5. Other diseases (differential diagnosis with PE) included pregnancy-induced hypertension (6 cases); intrauterine growth restriction with or without hypertension (4); chronic hypertension (6 cases); HELLP syndrome after delivery (1 case).

**Table 2:** Main outcome data.

Data at delivery (median; min–max or %)	CKD (*n*: 35)	PE (*n*: 24)	Other (*n*: 17)	Statistical significance
Cesarean section (%)	34.3%	75%	76.5%	*P*0 = 0.002
Gestational week	37 (30–40)	33 (27–38)	36 (33–39)	*P*0 < 0.001
Preterm (<37 weeks) (%)	40.0%	87.5%	58.8%	*P*0 = 0.001
Early preterm (<34 weeks) (%)	11.4%	54.2%	23.5%	*P*0 = 0.001
Weight at birth (grams)	2679 ± 610	1713 ± 710	2341 ± 664	*P*0 < 0.001
SGA <10% (INeS) (%)	5.7%	25.0%	29.4%	*P*0 = 0.048
SGA <5% (INeS) (%)	5.7%	16.7%	11.8%	*P*0 = 0.397

CKD: chronic kidney disease; PE: preeclampsia; SGA: small for gestational age baby, according to INeS charts. *P*0: significance across groups; *P*1: CKD versus PE; *P*2: CKD versus Other; *P*3: PE versus Other.

*Note*. Significance among groups: cesarean section: *P*1 = 0.007; *P*2 = 0.010; *P*3 = 1.0; gestational week: *P*1 < 0.001; *P*2 = 0.161; *P*3 = 0.003; preterm <37 weeks: *P*1 = 0.001; *P*2 = 0.327; *P*3 = 0.063; early preterm: *P*1 = 0.001; *P*2 = 0.413; *P*3 = 0.101; weight at birth (Bonferroni's Test): *P*1 < 0.001; *P*2 = 0.254; *P*3 = 0.010; SGA (<10°): *P*1 = 0.053; *P*2 = 0.031; *P*3 = 1.0.

**Table 3:** Molecular and biophysical biomarkers.

Biomarkers: median; min–max or %	CKD (*n*: 35)	PE (*n*: 24)	Other (*n*: 17)	Statistical significance
sFlt-1 (pg/mL)	1893 (585–13306)	11184 (308–28182)	5472 (222–15984)	*P*0 < 0.001

PlGF (pg/mL)	270 (11.5–1770)	39 (10.2–330.3)	94.4 (11.5–2564)	*P*0 < 0.001

sFlt-1/PlGF ratio	8.29 (1.04–137.40)	317.32 (28.05–2619.11)	58.96 (1.46–355.45)	*P*0 < 0.001

Normal umbilical and uterine flow	85.7%	37.5%	52.9%	*P*0 = 0.001
Normal uterine or umbilical flow	14.3%	29.2%	29.4%	
Abnormal umbilical and uterine flow	0%	33.3%	17.6%	

*P*0: CKD versus PE versus Other; *P*1: CKD versus PE; *P*2: CKD versus Other; *P*3: PE versus Other.

*Note*. Comparisons between groups: sFlt-1: *P*1 < 0.001; *P*2 = 0.004; *P*3 = 0.003. PlGF: *P*1 < 0.001; *P*2 = 0.002; *P*3 < 0.003. Ratio sFlt1-PlGF: *P*1 < 0.001; *P*2 = 0.001; *P*3 = 0.001; prevalence of normal flows: *P*1 < 0.001; *P*2 = 0.017; *P*3 = 0.508.

Presence of at least one impaired uteroplacental flow for diagnosis of PE and Other diseases versus CKD: sensitivity 56.1% (95% CI: 39.7%–71.5%); specificity 85.7% (95% CI: 69.7%–95.2%); PPV 82.1% (95% CI: 63.1%–93.9%); NPV value 62.5% (95% CI: 47.4%–76.0%).

PE only versus CKD: sensitivity 62.5% (95% CI: 40.6%–81.2%); specificity 85.7% (95% CI: 69.7%–95.2%); PPV 75% (95% CI: 50.9%–91.3%); NPV: 76.9% (95% CI: 60.7%–88.9%).

**Table 4:** Relationship between uteroplacental flow and the analyzed biomarkers.

	Normal umbilical and uterine flow (*n*: 48)	Normal uterine or umbilical flow (*n*: 17)	Abnormal umbilical and uterine flow (*n*: 11)	Statistical significance
sFlt-1 median (min–max)	2107 (308–16573)	8374 (222–25538)	16241 (2350–28182)	*P*0 < 0.001

PlGF median (min–max)	195.25 (10.98–2564.0)	42.77 (10.39–796.90)	41.07 (10.15–247.10)	*P*0 < 0.001

Ratio median (min–max)	9.94 (1.04–439.37)	136.26 (1.86–2457.94)	407.68 (9.51–2619.11)	*P*0 < 0.001

*P*0 = Kruskal Wallis; Mann Whitney *P*1 = normal versus partially altered flow; *P*2 = normal versus altered flow; *P*3 = partially altered flow versus altered flow.

sFlt-1: *P*1 = 0.011; *P*2 < 0.001; *P*3 = 0.025. PlGF: *P*1 = 0.017; *P*2 < 0.001; *P*3 = 0.264. Ratio PlGF/sFlt-1: *P*1 = 0.003; *P*2 < 0.001; *P*3 = 0.025.
